# Northern Schools and Southern Medicine

**Published:** 1845-03

**Authors:** 


### MEDICAL EXAMINER,

AND

### RECORD OF MEDICAL SCIENCE.

NEW SERIES—No. III.—MARCH, 1845.

---

### ORIGINAL COMMUNICATIONS.

The following communication has been handed to us by a distinguished physician, whoh as resided many years in the South, and had much experience in the treatment of its "*peculiar diseases.*" We are not surprised that he should be struck with the contrast between the views *formerly* advocated by his brethren in the South, and the opinions *now* entertained by them, and the similarity, almost identity, of the latter, with the doctrines taught for years past in some of the Northern Medical Schools. Truth and science are the same everywhere. EDITOR.

### NORTHERN SCHOOLS AND SOUTHERN MEDICINE.

TO PROFESSOR HUSTON:

SIR,—There is in the number of the Medical Examiner for November 2, 1844, a well conceived and well written article on the feeling—for it is evidently a matter of feeling rather than of judgment—which prevails with some, that in order for a physician to understand the principles of disease in any section of the country, he must have been educated there; notwithstanding, that the teachers in such section may adopt as text-books—even on the practice of physic—the productions of countries between which the wide Atlantic rolls. Very recently, I have noticed similar views expressed in a Southern Journal,—the *New Orleans Medical Journal* for 1844,—which appear to me to require some notice. And first,—as regards a communication from one of the Editors on the "Health of the Country," penned during, or in consequence of, a recent excursion up the Mississippi River to the States of Mississippi and Tennessee. "In regard to the diseases of the South," he says, "and especially our summer and autumnal fevers, we had conversations with numerous physicians of Louisiana, Mississippi and Tennessee, and found their views generally sound and correct. We venture to assert, that a more able and skilful set of practitioners is not to be found in any country. These men are not familiar with the minutiæ of Anatomy, Physiology and Pathology; their position renders this impossible; but they are well grounded in the general principles of medicine; they are men of close observation and sound judgment; above all, they are well acquainted with the *peculiarities* of the malignant diseases with which they have to contend, and act with promptness and skilful decision in cases, and under circumstances, that would confound the experience and scientific lore of a Chapman, Dunglison, Graves, Mackintosh, Bouillaud, Louis, Elliotson," &c. &c.

Let me then examine into the truth of this taunt, for which the writer will not, I apprehend,—since he has had time for reflection,—vindicate either the taste or the liberality. His object is clearly to show, that doctrines taught in the northern or other schools than those of the South and West, cannot apply to malignant diseases with "peculiarities" such as those that characterize the diseases of the Mississippi Valley.

Of the views taught in one of these schools—the Jefferson Medical College—I have ample knowledge from a careful attendance upon the lectures delivered in it; but I shall restrict myself here to the published opinions of one of the medical teachers alluded to in the above extract; and attempt to show, that principles taught upwards of "ten years ago," and still taught by him, are almost identical with those *now* embraced in the Southern portion of the Union.

Let me first cite the following remarks from the Journal, the comments of whose Editor have given occasion to the present communication.

"There has evidently," he says, "been a great alteration in the treatment of fevers among Southern practitioners within the last ten years. This change is to be remarked in two striking particulars: 1st, the more moderate use of emetics and purgatives; and 2d, the more prompt and bold administration of tonics; above all, the sulphate of quinine. The more frequent use of *topical bloodletting* may also be considered a modern improvement in this region. Formerly, a thorough evacuation of the entire alimentary canal was considered a *sine qua non* in the early stages of fever; and the most active medicines were freely given.

The liver, too, must be disgorged; and calomel was given with an unsparing hand. The consequence was, that often an incurable inflammation was established upon the mucous membrane of the stomach and bowels. But the views of practitioners are now almost wholly changed. Having observed that in openly developed Bilious Fevers, when death occurred, it was from the above named inflammation, or inflammation of the brain, they now avoid the use of irritating medicines, and endeavour to prevent or combat these formidable lesions by mild evacuants, venesection, cupping and leeching, and cold applications. As soon as a remission of the fever is procured, the *sheet-anchor, quinine*, is introduced, and the disease is soon cured. In cases of *congestive type*, which are often so insidious in their attacks as to deceive all but the *practised eye*, they lose no time in precursory preparation of the system, but resort at once to the *anti-periodic. Quinine*, instead of *Calomel*, is now considered in the South the *Sampson* of the *Materia Medica*; and we congratulate our brethren and the community on the change. The doses of this medicine have been increased from 2 grains up to 10, 20 and upwards; and its beneficial effects are often truly wonderful. Secretly and imperceptibly it dispels the morbid train of symptoms, and is perhaps the *only medicine* strictly entitled to the name of *Febrifuge*," &c. &c.

And again. "In regard to sanguineous depletion in the treatment of Bilious and Congestive Fevers, we found quite a diversity of opinion. Some eschew it altogether—some use topical bleeding early—and others practise venesection boldly, on the plan of Mackintosh in Intermittent Fevers."—So that the practitioners of these regions differ themselves, most essentially, in treatment, and consequently, in principles!

How different is the treatment extolled above, from that which was a few years ago universal, and which consisted in the lavish employment of the former "Sampson of the Materia Medica"—Calomel,—and against which certain Professors of the Northern schools protested most strenuously at the time it was so much in vogue.

"In all these cases," says Professor Dunglison in his "General Therapeutics," written upwards of "ten years" ago—"in which such large doses of calomel are administered, the practitioner is led to persuade himself that the climate requires them. But this argument is often most fallacious:—it may be employed, too, to bolster up any plan, that has received the approbation of a part or of most of the profession—too often, perhaps, without sufficient examination. It has been a common opinion, that in our ordinary bilious fevers, copious bloodletting, and the most active and irritating cathartics, are imperiously demanded; and the practice founded upon this belief was at one time universal; so much so, indeed, that no other has been adopted extensively until of late years; but since a greater degree of attention has been paid to the condition of the mucous membrane in these affections, and since a better philosophy has suggested, that whilst we are keeping the different external sensitive surfaces free from all irritation, we ought not to be perpetually irritating the internal dermoid prolongation, practitioners have been induced to abandon the use of irritating cathartics,—to keep the digestive canal free by the use of mild cathartics, which remove the morbid secretions as they are formed; and, by the proper use of sedatives—of which bloodletting is almost the only one—and of refrigerants, to reduce the inflammatory excitement. By such a plan—and we can equally invoke *experience* in its support—the ordinary bilious fevers of our country will be found to yield far more satisfactorily than under the mixed sedative and irritating treatment, which was formerly universal, and still prevails too extensively. It is obvious, too, that where one system of medication is exclusively employed, it is impossible to draw any deductions from comparison, and we are not justified in affirming that climate requires one system more than another, until an equal trial has been made of all."

Yet, a few years ago, it was considered to be heretical for any one to raise objections against the use of the then "Sampson article;" whilst at this day we are told, that "*Quinine*, instead of *Calomel* is now considered in the South the Sampson of the Materia Medica; and we congratulate our brethren and the community on the change:"—to which I cordially respond, *Amen!*

In a subsequent section, speaking of the use of Tonics, Professor Dunglison remarks—"There are cases, however, of remittent fever occurring in highly malarious districts, which demand the use of the bark comparatively early;—the disease from the first not exhibiting any highly phlogistic character, and being apt to be attended with engorgements of internal organs, unless interrupted in its progress at an early period."

These views—let it be recollected—were maintained upwards of "ten years ago," and when the perturbating calomel treatment was in full ascendancy; and they were not merely theoretical, but deduced from the observation and experience of the author in a Southern State. Since that period, they have been more strongly urged in his "Practice of Medicine" and other works, and in the Clinical Lectures delivered at the Philadelphia Hospital, reports of which have been published during the last two clinical sessions in the pages of the Examiner.

It might be regarded as a vague statement to refer, as I could do, to lectures of Professors in the Northern Schools, enforcing the same views, which have never been published, and hence I have brought forward only opinions that have been for sometime before the Profession, and can be readily turned to.

To indicate still more how the tide has set against the indiscriminate use of calomel, I may refer to an article, in the same New Orleans Journal, on Congestive Fever, by Dr. Monette, of Washington, Mississippi, which exhibits attentive observation and much reflection, with too many indications, however, of the prevalent feeling of the South, in regard to Northern teaching, which have as little foundation as those already animadverted upon.

"For nearly seven years," he says, "I have been convinced that there was a portion of our summer and autumnal diseases, which were aggravated and protracted by the use of calomel; and during that time I have often been induced to lay aside the use of calomel, and substitute a soothing practice, in many cases where calomel had proved decidedly injurious. It is more than fifteen years, since I first observed that calomel was *literally a poison* in those cases which were attended with great *irritability of the liver and portal circle.* I had seen almost every yellow fever patient die, who had been treated under the full mercurial course; besides many cases of common bilious remittent fever. I had observed plainly, that there were two *classes of bilious and remittent fevers;* that one was attended with functional torpor of the liver, and the other with a morbid irritability of the same organ; and that one was benefited by the administration of calomel, and the other was aggravated by the same treatment, but yielded under a judicious course of anodyne and refrigerant practice. Under this conviction, I had in some cases ventured, contrary to all my former opinions, and to the highest authority, to place my chief reliance upon salines and narcotic remedies, and such articles as were calculated to *allay intestinal irritation.* Still I could not entirely dispense with the sheet-anchor, the Sampson of the Materia Medica,"—[*now*, it will be recollected in the opinion of the Editor of the New Orleans Journal, the sulphate of quinia, not calomel,] "which I still considered necessary in the first onset of the treatment; although it might be necessary to discontinue it afterwards. Yet it was not until the year 1843, that I learned by actual experiment, that good bilious discharges and speedy salutary termination of *severe cases* of bilious and congestive fevers, could be obtained more readily without calomel than with it. Such was my faith in the efficacy of calomel, in inducing a salutary secretion from the liver, in such cases, that it was long before I could *venture* to risk the life of my patient to any course of treatment which excluded calomel. And in cases of great danger, where calomel appeared to exert *no salutary influence,* it was with fear and trembling, that I ventured to treat the case by substituting other remedies for this formerly indispensable article. At length in the spring of 1843, I ventured to attempt the treatment of several cases to the entire exclusion of calomel, and the result was most flattering and eminently successful."

In other words, in 1843, Dr. Monette, ventured to put in practice principles which had been long inculcated in at least one of the Northern Schools, and most successfully carried out by many of the pupils of that School in the South and West. Of the fact, however, of such inculcations, he was, doubtless, as little informed as he is of the doctrines maintained in the same school on the subject of *duodenal irritation!*" "The extensive influence"—he observes—"exerted over the whole system, by morbid conditions of the *duodenum,* in some other diseases" [than Congestive Fever,] "has been too often overlooked, or disregarded by practitioners in the South, who have received their education and their prejudices in the Northern Schools. [!!] In tropical latitudes, such as the State of Louisiana, and the Southern half of the States of Mississippi and Alabama, this organ is more frequently implicated in our chronic affections, and in our violent, rapid and fatal summer diseases, than most practitioners apprehend ; but in none, more than in Congestive Fever, and the malignant Yellow Fever of Tropical America. Ignorance or a disregard of this fact, as well as ignorance of the peculiar *morbid sensibility* of this organ, when irritated, to certain medicines, is one cause of much of the fatality attending such diseases. An intimate knowledge of this peculiar sensibility of the duodenal surfaces, when irritated, to certain medicinal substances, would certainly deter practitioners from the excessive use of such as are peculiarly obnoxious to this state of the duodenum."

It is obvious from the whole of his remarks on this matter, that Dr. Monette is not only unacquainted with the views in regard to bilious affections taught in "Northern Schools," but even with those contained in works emanating from those schools, which are notwithstanding extensively diffused over the country. The principles laid down by Dr. Monette are, indeed, essentially those of Broussais, and of every one who has observed and reflected deeply on the subject.

"One of the most important circumstances," says Professor Dunglison, "to be borne in mind in the history of remittent fever, is the tendency to local hyperæmia or inflammation; the presence of which gives the peculiar characters, by which we find the remittent of one season distinguished from that of another. In all cases, perhaps, the lining membrane of the stomach or small intestines, or both, is implicated; and hence the term

*gastric* so universally applied to it. In hot climates and seasons, an unusual degree of erethism exists in the gastro-enteric mucous membrane, which, under the influence of the causes of remittent fever, and the irregular actions occurring in the course of the disease, may be developed into inflammation of greater or less severity. Where it is to a great degree, it spreads along the biliary ducts to the liver; for a time arrests the secretion of the bile, and gives occasion to functional phenomena denoting hepatic complication,—such as pain and sense of fulness in the right hypochondrium; absence of bile in the matters evacuated by vomiting, and in the alvine evacuations; with yellowness of the surface of the body, and especially of the tunica conjunctiva. After awhile, however, in favorable cases, the hyperæmic or inflamed condition of the liver passes away, and the restoration of the hepatic functions is denoted by the evacuation of a dark pitch-like matter, which has been esteemed *critical*, but probably denotes nothing more than that the pathological cause of the deficient biliary secretion has passed away, and that the engorgement, which was the cause of the detention of the bile in the gall-bladder and ducts, had subsided. In other cases where the gastro-enteric hyperæmia is not so great, the hepatic symptoms may be less marked, or be indicated for the first few days simply by a yellowish tinge of the conjunctiva, denoting that the secretion is not freely poured into the small intestine. The liver, in this case, instead of having its secretion locked up, as it were, in the gall-bladder and ducts, after the first few days is merely excited to greater secretion, and, accordingly, the disease is accompanied by a copious flow of bile, which is indicated in the evacuations both by vomiting and stool. According, therefore, to the degree of gastro-enteritis existing in any individual case, there may be signs of absence, or of undue quantity of bile in the evacuations; but in both cases, the liver is affected secondarily,—a slight irritation in the lining membrane of the duodenum acting in the same manner as one of our cholagogne cathartics; the irritation produced by it being communicated, in the manner above mentioned, along the ductus communis choledochus, and the hepatic duct to the liver, and along the cystic duct to the gall-bladder, so that the former is excited to greater activity of secretion, and the latter to a more frequent discharge of its contents." (*Practice of Medicine*, second edition, Vol. 2, p. 438.)

The above remarks are sufficient to show—what ought not to be contested—that principles of medicine belong not exclusively to any locality, but are of universal application; and that they may be sought after with at least as much certainty and safety in the Northern, as in the Southern portions of our continent; and whilst it must be admitted, that locality and epidemic influences may interfere, more or less, with the nature and manifestations of disease, it must be equally admitted, that the physician, who is well grounded in principles, will readily seize hold of the differences, and apply his therapeutical agents with full understanding. As an inhabitant of a southern state, who has visited a Northern School for the purpose of reaping the advantages which such schools afford in enabling the student to become well grounded in the principles and practice of his profession, I have considered it but a duty to bring forth evidence, which may enable my southern friends to satisfy themselves, that erroneous notions have prevailed on the subject, and that whilst it has been believed by some that there is much discrepancy between the profession here and in the South and West on many points, connected with the pathology and treatment of the diseases of those localities, the fact is, that amongst the best informed there is a signal similarity, if not identity of sentiment.

*February* 1, 1845,

---

*Contribution to the History of Malaria. In a letter from* Dr. R. S. Holmes, *of the Medical Staff of the United States Army, to* Professor Dunglison. (Communicated by the latter.)

*Philadelphia*, November 23d, 1844.

To Professor Dunglison:

